# Supplementary material for: Developing a typology of models of palliative care delivery in prisons in high-income countries: protocol for a scoping review with narrative synthesis
Source: BMJ Open. 2022 Apr 29;12(4):e060886. doi: 10.1136/bmjopen-2022-060886 (PMC9058786; doi:10.1136/bmjopen-2022-060886)
Supplement: Supplementary data [file bmjopen-2022-060886supp001.pdf]

Supplementary Material – MEDLINE search strategy

The preliminary search parameters are English-only published research papers, commentaries and discussions, reviews and policy documents concerning palliative and end-of- life care for the prison population.

#	Query
1	Palliative Care/
2	Terminal Care/
3	Death/
4	Terminally Ill/
5	Hospice Care/
6	Pain Management/
7	Advance Directives/
8	Advance Care Planning/
10	Palliat*.mp.
11	end of life.mp.
12	end of life care.mp.
13	terminal illness.mp.
14	dying.mp.
15	end stage illness.mp.
16	supportive care.mp.
17	symptom management.mp.
18	compassionate release.mp.
19	1 or 2 or 3 or 4 or 5 or 6 or 7 or 8 or 9 or 10 or 11 or 12 or 13 or 14 or 15 or 16 or 17 or 18
20	Prisoners/ or Prisons/
21	Criminals/
22	Jails/
23	Incarcerat*mp.
23	Correctional Facilities/
24	convict*.mp.
25	felon*.mp.
26	offender*.mp.
27	inmate*.mp.
28	penitentiar*.mp. [
29	gaol.mp.
30	secure.mp.

31	20 or 21 or 22 or 23 or 24 or 25 or 26 or 27 or 28 or 29 or 30
32	19 and 31
