# Supplementary material for: Developing a typology of models of palliative care delivery in prisons in high-income countries: protocol for a scoping review with narrative synthesis
Source: BMJ Open. 2022 Apr 29;12(4):e060886. doi: 10.1136/bmjopen-2022-060886 (PMC9058786; doi:10.1136/bmjopen-2022-060886)
Supplement: Supplementary data [file bmjopen-2022-060886supp002.pdf]

CINAHL search strategy	
S55	S40 AND S54.
S54	S41 OR S42 OR S43 OR S44 OR S45 OR S46 OR S47 OR S48 OR S49 OR S50 OR S51 OR S52 OR S53)
S53	“secure”
S52	“gaol”
S51	“penitentiar*”
S50	“inmate”
S49	“offender”
S48	“felon”
S47	“convict*”
S46	(MH “Correctional Facilities”)
S45	“icarcerat*”
S44	“Jail*”
S43	"Criminal*"
S42	(MH "Prisoners")
S41	"Prison*"
S40	S21 OR S22 OR S23 OR S24 OR S25 OR S26 OR S27 OR S28 OR S29 OR S30 OR S31 OR S32 OR S33 OR S34 OR S35 OR S36 OR S37 OR S38 OR S39
S39	"'compassionate release'"
S38	"'symptom management'"
S37	"'supportive care'"
S36	"'end stage illness'"
S35	"dying"
S34	dying
S33	"terminal illness"
S32	"'end-of-life care'"
S31	"'end-of-life'"
S30	"Palliat*"
S29	(MH "Advance Care Planning")
S28	(MH "Advance Directives+")

S27	(MH "Pain Management")
S26	(MH "Hospice Care")
S25	Terminally ill
S24	(MH "Terminally Ill Patients+")
S23	(MH "Death+")
S22	(MH "Terminal Care+")
S21	(MH "Palliative Care")

EMBASE search strategy	
#	Query
1	exp palliative therapy/
2	exp terminal care/
3	exp death/
4	exp terminally ill patient/
5	exp hospice care/
6	"pain management".mp.
7	'advance directives'.mp.
8	exp advance care planning/
9	Palliat*.mp.
10	"end-of-life".mp.
11	"end-of-life care".mp.
12	exp terminal disease/
13	"terminal illness".mp.
14	exp dying/
15	"end stage illness".mp.
16	"supportive care".mp.
17	"symptom management".mp.
18	"compassionate release".mp.

19	Palliative care.mp.
20	Terminal care.mp.
21	death.mp.
22	terminally ill.mp.
23	hospice care.mp.
24	exp hospice/
25	advance care planning.mp. or advance care planning/
26	dying.mp.
27	prisoner/ or prison/
28	prison.mp.
29	prisoner.mp.
30	Criminal*.mp.
31	Jail*.mp.
32	Incarcerat*.mp.
33	correctional facility/
34	exp correctional facility/
35	convict*.mp.
36	felon*.mp.
37	exp offender/
38	offender.mp.
39	inmate*.mp.
40	penitentiar*.mp.
41	gaol*.mp.
42	1 or 2 or 3 or 4 or 5 or 6 or 7 or 8 or 9 or 10 or 11 or 12 or 13 or 14 or 15 or 16 or 17 or 18 or 19 or 20 or 21 or 22 or 23 or 24 or 25 or 26

43	27 or 28 or 29 or 30 or 31 or 32 or 33 or 34 or 35 or 36 or 37 or 38 or 39 or 40 or 41
44	42 and 43
45	limit 44 to yr="2000 -Current"

**Social Sciences Citation Index Search Strategy**
**#9 AND**
**#10** and **2000** or **2001** or **2002** or **2003** or **2004** or **2005** or **2022** or **2021** or **2020** or **2019** or **2018** or **2017** or **2016** or **2015** or **2014** or **2013** or **2012** or **2011** or **2010** or **2009** or **2008** or **2007** or **2006** (Publication Years)
**Prion* OR Prisoner* OR Jail* OR Incarcerat* OR "Correctional Facilities" OR Convict* OR felon* OR offender* OR inmate* OR penitentiar* OR gaol** (Topic)
**"Palliative Care" OR "Terminal Care" OR Death OR '"Terminally ill" OR "Hospice Care" OR "Pain Management" OR "Advance Directive*"OR "Advance Care Planning" OR Palliat* OR "end-of-life" OR "terminal illness" OR dying OR "end stage illness" OR "supportive care" OR "symptom management" OR "compassionate release"** (Topic)
**#5 AND #6**
**TS=(Prion* OR Prisoner* OR Criminal* OR Jail* OR Incarcerat* OR "Correctional Facilities" OR Convict* OR felon* OR offender* OR inmate* OR penitentiar* OR gaol OR secure)**
**TS=("Palliative Care" OR "Terminal Care" OR Death OR '"Terminally ill" OR "Hospice Care" OR "Pain Management" OR "Advance Directive*"OR "Advance Care Planning" OR Palliat* OR "end-of-life" OR "terminal illness" OR dying OR "end stage illness" OR "supportive care" OR "symptom management" OR "compassionate release")**
**#1 AND**<br>**#2** and **2000** or **2001** or **2002** or **2003** or **2004** or **2005** or **2006** or **2007** or **2008** or **2009** or **2010** or **2011** or **2012** or **2013** or **2014** or **2015** or **2016** or **2017** or **2018** or **2019** or **2020** or **2021** or **2022** (Publication Years)
**#1 AND #2**

**ALL=(Prion* OR Prisoner* OR Criminal* OR Jail* OR Incarcerat* OR 'Correctional Facilities' OR Convict* OR felon* OR offender* OR inmate* OR penitentiar* OR gaol OR secure)**
**TS=('Palliative Care' OR 'Terminal Care' OR Death OR 'Terminally ill' OR 'Hospice Care' OR 'Pain Management' OR 'Advance Directive*' OR 'Advance Care Planning' OR Palliat* OR 'end-of-life' OR 'terminal illness' OR dying OR 'end stage illness' OR 'supportive care' OR 'symptom management' OR 'compassionate release')**

**PsyINFO Search Strategy**	
1.	Palliative care.mp. or exp palliative therapy/
2.	Terminal Care.mp. or exp terminal care/
3.	exp death/ or Death.mp.
4.	Terminally ill.mp. or exp terminally ill patient/
5.	Hospice Care.mp. or exp hospice care/
6.	'Pain management'.mp.
7.	'Advance* Directive*'.mp.
8.	'Advance* Care Planning'.mp. or exp advance care planning/
9.	Palliat*.mp.
10.	'end of life'.mp.
11.	'end of life care'.mp.
12.	'terminal illness'.mp. or exp terminal disease/
13.	dying.mp. or exp dying/
14.	'end stage illness'.mp.
15.	'supportive care'.mp.
16.	'symptom management'.mp.
17.	'compassionate release'.mp.
18.	1 or 2 or 3 or 4 or 5 or 6 or 7 or 8 or 9 or 10 or 11 or 12 or 13 or 14 or 15 or 16 or 17
19.	Prison*.mp. or exp prison/ or exp prison nursing/ or exp prisoner/
20.	Criminal*.mp. or exp criminal justice/ or exp criminal behavior/

21.	Jail*.mp.
22.	exp incarceration/ or Incarcerat*.mp.
23.	'Correctional Facilities'.mp. or exp correctional facility/
24.	convict*.mp.
25.	felon*.mp.
26.	exp offender/ or offender*.mp.
27.	inmate*.mp.
28.	penitentiar*.mp.
29.	gaol*.mp.
30.	secure.mp.
31.	19 or 20 or 21 or 22 or 23 or 24 or 25 or 26 or 27 or 28 or 29 or 30
32.	18 and 31
