# Supplementary material for: Developing a typology of models of palliative care delivery in prisons in high-income countries: protocol for a scoping review with narrative synthesis
Source: BMJ Open. 2022 Apr 29;12(4):e060886. doi: 10.1136/bmjopen-2022-060886 (PMC9058786; doi:10.1136/bmjopen-2022-060886)
Supplement: Author's manuscript [file bmjopen-2022-060886.draft_revisions.pdf]

BMJ Open

BMJ Open is committed to open peer review. As part of this commitment we make the peer review history of every article we publish publicly available.

When an article is published we post the peer reviewers' comments and the authors' responses online. We also post the versions of the paper that were used during peer review. These are the versions that the peer review comments apply to.

The versions of the paper that follow are the versions that were submitted during the peer review process. They are not the versions of record or the final published versions. They should not be cited or distributed as the published version of this manuscript.

BMJ Open is an open access journal and the full, final, typeset and author-corrected version of record of the manuscript is available on our site with no access controls, subscription charges or pay-per-view fees (http://bmjopen.bmj.com).

If you have any questions on BMJ Open's open peer review process please email info.bmjopen@bmj.com

BMJ Open

Developing a typology of models of palliative care delivery in prisons in high income countries: protocol for a scoping review with narrative synthesis

Journal:	*BMJ Open*
Manuscript ID	bmjopen-2022-060886
Article Type:	Protocol
Date Submitted by the Author:	10-Jan-2022

Complete List of Authors:	GILBERT, Emma; University of Bristol<br>Turner, M; University of Huddersfield,<br>de Viggiani, Nick; University of the West of England Bristol,<br>Selman, Lucy; University of Bristol School of Social and Community Medicine

Keywords:	PALLIATIVE CARE, Adult palliative care < PALLIATIVE CARE, HEALTH SERVICES ADMINISTRATION & MANAGEMENT

SCHOLARONE™ Manuscripts

Developing a typology of models of palliative care delivery in prisons in high income countries: protocol for a scoping review with narrative synthesis

Emma Gilbert[1,2], Mary Turner[3], Nick De Viggiani[4], Lucy E. Selman[1]

[1]Palliative and End of Life Care Research Group, Bristol Medical School, University of Bristol, UK

[2]National Institute of Health Research

[3]NHS England, Health & Justice, South-West

Corresponding author: Emma Gilbert emma.gilbert@bristol.ac.uk

For peer review only

Abstract

Introduction

A combination of punitive sentencing practices within ageing populations, compounded by the health challenges faced by prisoners, mean that dedicated palliative care provision within prisons is a pressing requirement. However, evidence about exactly how quality palliative and end-of-life care is delivered in this environment remains sparse.

This review aims to develop a typology of models of palliative and end-of-life care delivery within prisons in high-income countries to inform service development and policy.

Methods and Analaysis

We will conduct a scoping review of published studies and grey literature, following the Arksey & O'Malley framework (1). Data will be reported. We will report data on models of palliative and end-of-life care delivery in prisons in high-income countries. Searches will be undertaken in Medline, EMBASE, CINAHL, Social Sciences Citation Index and Psych Info for all study types, published from 1st January 2000 to December 2021, and reference lists from key reviews and studies will be screened for additional references. We will also screen grey literature from within other high-income countries using a targeted search strategy. For published reports of original research, study quality and risk of bias will be assessed independently by two reviewers using the Mixed Methods Appraisal Tool. A narrative synthesis of the data will be undertaken, integrating the results of the quality assessment.

Ethics and dissemination

Approval by research ethics committee is not required since the review only includes published and publicly accessible data. We will publish our findings in a peer-reviewed journal as per PRISMA 2020 guidance.

For peer review only

Protocol registration

The final protocol was registered with the Research Registry on 26.11.2021 www.researchregistry.com

Unique ID number: reviewregistry1260

Strengths and limitations of this study

- This protocol conforms to the Preferred Reporting for Scoping reviews extension (PRISMA) 2020 guidelines.
- The interpretation of 'models of care' escapes clear definition within the research literature, so it is not possible to include it in the search strategy; this information will be extracted, and a typology developed using a pre-piloted data extraction template.
- We adopt a narrative synthesis approach as initial searches suggest that the studies identified will be insufficiently similar in research design and there will be a high volume of grey literature such as policy documents and statutory reports.
- Narrative synthesis will provide an in-depth understanding of the literature on how palliative care is delivered in prisons across high income countries, informing subsequent research.

For peer review only

Introduction

This review intends to develop a typology of models of palliative care delivery within prisons in high-income countries. A combination of punitive sentencing practices within ageing populations, compounded by the health challenges faced by prisoners, mean that dedicated palliative care provision is a pressing requirement within many prisons (2), (3). However, evidence about exactly how quality end of life care is delivered in these environments remains sparse (2).

With the largest prison population in Western Europe, the older prisoner demographic is growing rapidly within England and Wales (prisons are devolved within the UK). People aged 60 and over are the fastest growing age group in the prison estate, which is three times as many as sixteen years ago (4). This trend is visible across Europe - of the 48 prison administrations providing data for the Council of Europe's 2020 SPACE report on prison indicators, 14.8% of inmates were aged 50 or over. Imprisoned individuals living behind bars now represent the fastest growing group in correctional facilities in the UK, and also Australia, Switzerland, Japan and the USA (Petreca 2021). Table 1 illustrates the percentage increase of older age prisoners across high-income countries, and the number of older prisoners is expected to increase significantly in coming years (5).

Table 1: Percentage increase of older age prisoners across high income countries

Date range	Country	Age	Percentage Increase	Source of data
2013 -2018	Singapore	60 +	50 %	(22)
2013-2018	S Korea	65 +	45%	(22)
2007-2017	Switzerland	50 +	100%	(22)
1990-2030	USA	55 +	4400%	(22)

For peer review only

2010-2019	Canada	50 +	50%	(22)
2002-2020	UK	60 +	243%	(23)
2000-2010	Australia	65+	84%	(17)
2000-2009	New Zealand	50+	94%	(24)

There is evidence of an association between incarceration and poor health outcomes (6). Prisons tend to accumulate individuals who have experienced significant health inequalities, with far greater incidences of mental health and substance misuse disorders, as well as physical health co-morbidities, than the general population (3). These health disparities are often intensified by the environmental challenges of delivering healthcare within the built environment. Ageing buildings which cannot ensure rigorous infection prevention control, cells that lack adequate space for specialist equipment and a regime that imposes limitations on an individual's self-efficacy in their own nutrition, physical activity, relaxation and sleep inevitably affects an individual's ability to cope (7).

Prisoners consequently face increased morbidity (8). In a 2018 rapid review, the estimated annual prevalence of those requiring end-of-life care in French prisons was twice as high as the anticipated equivalent expected in the general population, and comparable to a population 10 years' older (8).

Research into palliative care within the penal system is an emerging area, and substantial gaps remain regarding the current nature of provision and best practice models. Recent investigation by the European Association for Palliative Care Task Force for Prisoners addressed some of these through data collection within eight countries, examining palliative care provision, causes of death in custody and the application of early release on compassionate grounds policies (2). This research highlighted the inequitable provision for those either dying or living with a life-limiting

For peer review only

illness in prison, as well as the limited potential that current early release policies offer in practice (2). Other salient research has focused on the ethical challenges that delivering palliative care within a human rights framework poses within the prison system (9) and the experience of facing terminal illness whilst incarcerated (10), as well as the 'double burden' experienced by older prisoners who face the deprivation of liberty that is standard amongst those incarcerated, but are subject to additional suffering by the failure of prison healthcare systems to adequately meet their needs (11).

In the United Kingdom, understanding the palliative and end-of-life care needs for prisoners has gained traction and many prisons have well-coordinated relationships with their local palliative care teams and hospices (12) The publication of the Dying Well in Custody Charter - End-of-Life Care Ambitions (13)articulated these developments as a set of standards for end-of-life care in prisons, but there is variation in how the Charter has been applied.

This review extends this work in significant ways, by comprehensively mapping, for the first time, models of palliative healthcare delivery for those facing a life-limiting illness within prisons across high-income countries and synthesizing related evidence. Findings from the review will help to ensure that best available evidence informs future provision of culturally relevant, tailored support, as well as providing a basis for policy making regarding, for example, Early Release on Compassionate Grounds and alternative secure accommodation for ageing prisoners.

In accordance with guidelines, our scoping review protocol was registered with the Research Registry on the 26/11/2021 [ID: xxxxxxxx].

Aim

This scoping review aims to map and synthesize the literature on models of palliative and end-of-life care for prisoners, within prisons in high-income countries. Its objectives are to describe models of service delivery that currently exist in published and grey literature, appraise these models in terms of outcomes and impact, and

For peer review only - http://bmjopen.bmj.com/site/about/guidelines.xhtml

describe facilitators of and the challenges in delivering different models of palliative and end-of-life care for prisoners. The synthesis will then consider how the identified models meet the overall intentions of palliative care, drawing out implications and recommendations for service provision and policy.

Review questions

SPICE framework

Setting – adult prisons both male and female

Perspective – prison staff, prison volunteers, patients and their family/carers (who have current or prior experience of a family member receiving end of life care in prison).

Intervention – model of palliative care/ end-of-life healthcare delivery - be it a prison hospice, specialist in-reach palliative care provision to a prison, or another integrated model

Comparison - qualitative and mixed methods studies are unlikely to have a comparison group; quantitative studies may compare the intervention with usual care or with a comparison/control group

Evaluation – mapping and describing available models of palliative healthcare delivery in terms of acceptability and usefulness to patients, family/carers and clinicians; outcomes and impact of these models; and facilitators of their implementation.

Primary question: What models of palliative and end-of-life care for prisoners are described in both the published and grey literature?

Secondary questions:

What evidence exists regarding the outcomes and impact of these models?

What are the facilitators of and challenges in delivering different models of palliative and end-of-life care for prisoners?

Methods

For peer review only

Inclusion criteria

Study reports will be included in this scoping review if they meet the following inclusion criteria:

1. Any study reporting new empirical data, regardless of study design.
2. Studies reporting models and mechanisms of palliative and end-of-life healthcare delivery to the prison population within other high-income countries.
3. Studies reporting the views and experiences of different models of palliative and end-of-life healthcare delivery in prison from the perspective of:
    a. Prisoners, their families and informal carers (including in bereavement)
    b. Prison staff and volunteers
4. Studies conducted in high-income countries that are published in English. High-income countries are defined by the World Bank as having a gross national income per capita exceeding $12,056 (14).
5. Studies reported since 1st January 2000.

Exclusion criteria

1. Studies not reported in English.
2. Studies reporting on chronic or life-limiting illness, death and dying within prisoner and criminal justice contexts where the model of care delivery is not described.
3. Studies about institutions that do not fall under the legal definition of prison (such as Immigration Removal Centres), or do not cater for adult prisoners (Secure Children's Homes).
4. Studies about prison palliative care, where patient/caregiver or staff experiences are reported, but the model of care delivery is not described or evaluated.
5. Studies that focus on components of palliative care provided at specific phases of the disease trajectory and do not describe the overall model of palliative care delivery (e.g., pain management only)
6. Studies from low and middle-income countries.
7. Studies published prior to 1st January 2000.

For peer review only

Grey literature such as conference abstracts, audits, theses and dissertations, research and committee reports, government reports, policy documents, quality improvement reports and ongoing research will be included if they present relevant empirical data. If there is uncertainty about whether the inclusion criteria are met, or if relevant data cannot be extracted, the authors will be contacted to ask if they can provide additional information and/or further data. If this is not possible the study will be excluded.

Adopting the five stages of the Arksey & O'Malley framework as shown in table 2 (1), this review aims to identify all relevant literature available on the topic, regardless of study design. This method is especially advantageous for assembling emerging evidence, as well as being suitable for addressing questions that go beyond the scope of effectiveness of an intervention (15). The approach adopts an iterative process of study selection, data collation, synthesis and presentation (16).

Table 2 – Five stages of the Arksey & O'Malley scoping review

Stage of review	Illustration of decisions and issues
Identifying the **research** question	Theoretical and empirical work describing models of palliative care delivery to prisoners in other high-income countries, with broadly comparable criminal justice systems and approaches to human rights.<br><br>Greater understanding of best practice, challenges and barriers to access
Identifying relevant **studies**	Specific search criteria designed with key terms used included palliative care, hospice, end-of-life, compassionate release, prison, penitentiary, imprisonment, incarceration, jail, custody, advanced care planning.

For peer review only

**Study** selection	Final included studies may include a diverse representation of primary sources; data will be extracted using the JBI Mixed Methods Data Extraction Form following a Convergent Integrated Approach
Charting the data	Data will be extracted from primary sources, different models of care summarised, best practice and barriers to access identified in a narrative synthesis
Collating, summarizing and reporting the results	Recommended models proposed with areas for further research and development identified

This review will build on the five stages of the Arksey & O'Malley framework by including critical appraisal of the quality of published studies, using the Mixed Methods Appraisal Tool (MMAT), version 2018 (17).

Search strategy

The following databases will be searched for English language studies:

Medline and EMBASE in Ovid, CINAHL, the Social Sciences Citation Index and PsychInfo.

Additional hand searches of key journals, screening of reference lists of included studies, citation tracking and input from expert collaborators will supplement the database searches. A further exploration of the grey literature will be conducted through searches of key websites (e.g., International Association for Hospice and Palliative Care, the WHO) and key grey literature databases (ProQuest, Google).

For peer review only

The Medline search strategy is shown in Supplementary Material. This strategy will be adapted to the other electronic databases, with any modifications reported in the review manuscript. Databases searches were run on 13th October 2021. The expected end date for the review is 30th April 2021.

Screening and data extraction

Search results from each database will be downloaded and managed in Covidence, an online review management platform (Covidence Systematic Review Software).

Each title and abstract will be screened against the inclusion/exclusion criteria by one of the review team. A second reviewer will independently screen a sample of 25% of the titles and abstracts. Full text will be reviewed if a reviewer is unclear based on title and abstract. Any discrepancies of study inclusion will be adjudicated by a third reviewer. Grey literature will be screened and synthesised separately and will not be subject to the same method of quality appraisal.

EG will extract data using a pre-piloted, customised data extraction form based on the JBI Mixed Methods Data Extraction Form following a Convergent Integrated Approach (18), in Covidence. Data extraction will be reviewed by a second reviewer and modified where needed. Discrepancies regarding data extraction will be resolved by discussion and consensus, and if necessary, include a third reviewer.

Quality assessment

The quality of all included studies published in peer reviewed journals will be assessed independently by two reviewers using the Mixed Methods Appraisal Tool (MMAT), version 2018 (17). This validated tool is appropriate for this review as it can be applied to qualitative, quantitative (randomised, non-randomised, and descriptive), and mixed-methods study designs. The tool uses a set of questions specific to study design, converted into four possible scores (worst to best: 25/50/75/100). Disagreements between the reviewers will be resolved through discussion, involving a third reviewer if necessary. No studies will be excluded based on their quality, but the narrative synthesis will reflect on the quality of the identified studies. Grey literature will not be subject to quality appraisal and will be analysed and reported separately.

For peer review only

Evidence synthesis

A narrative synthesis will be conducted to synthesize the findings of the different studies. Due to the potential range of studies that may be included in this integrative review, a narrative synthesis is the most appropriate way to synthesise the findings.

This review will follow the narrative synthesis approach outlined by Popay et al (2006). This process will involve developing a preliminary synthesis, exploring relationships within and between the studies, and assessing the robustness of the synthesis overall (19)

Grey literature will be synthesised and described where it adds relevant data to the research topic. The narrative synthesis will move beyond simply summarising the main features of included studies, presenting the data in such a way that it enables investigations into similarities and differences between studies, whilst assessing the data and strength of the evidence (20) The synthesis will be structured around the core models of palliative and end-of-life care delivery for prisoners. For each model, the following data will be synthesised: effectiveness and impact, facilitators for implementation, challenges and barriers of implementation. Implications for future service delivery, policy and research will be identified.

Patient and Public Involvement

No patient involved

Ethics and dissemination

This scoping review of published/publicly available studies is exempt from ethical approval. The review will be reported as per PRISMA guidance (21), and published in a peer-reviewed journal.

Contributors

For peer review only

EG initiated and designed the review as part of an NIHR pre-doctoral fellowship supervised by LES and NdV. LES, MT and NdV contributed to the design of the protocol. EG drafted the manuscript. All the authors contributed to the revision of the manuscript and approved the final version.

Acknowledgements

The authors would like to thank Sarah Herring, University of Bristol Subject Librarian, for support in developing the database searches and Isla Kuhn, Head of Medical Library Services, University of Cambridge Medical Library for support in developing the grey literature search strategy.

Funding

This work is undertaken as part of EG's National Institute for Health Research (NIHR) Pre-doctoral Fellowship. LES is funded by an NIHR Career Development Fellowship. The views expressed in this publication are those of the authors and not necessarily those of the NHS or the NIHR.

Competing interests

None declared.

Provenance and peer review

Not commissioned, externally peer reviewed.

Keywords

Palliative care, Hospice Care, End-of-Life care, Prisoners, Prisons.

For peer review only

References

1. Arksey H, O'Malley L. scoping. International journal of social research methodology. 2005;8(1):19–32.
2. Turner M, Chassagne A, Capelas ML, Chambaere K, Panozzo S, Teves CM, et al. Mapping palliative care provision in European prisons: an EAPC Task Force Survey. BMJ Supportive & Palliative Care [Internet]. 2021 Apr 22 [cited 2021 Apr 29]; Available from: https://spcare.bmj.com/content/early/2021/04/21/bmjspcare-2020-002701
3. Stürup-Toft S, O'Moore EJ, Plugge EH. Looking behind the bars: emerging health issues for people in prison. British Medical Bulletin [Internet]. 2018 Mar 1;125(1):15–23. Available from: https://doi.org/10.1093/bmb/ldx052
4. Prison Reform Trusts. Prison: the facts Bromley Briefings Summer 2019 [Internet]. 2019. 1987 [cited 2022 Jan 3]. Available from: http://www.prisonreformtrust.org.uk/Portals/0/Documents/Bromley%20Briefings/Prison%20the%20facts%20Summer%202019.pdf
5. Kratcoski PC, Edelbacher M, Kratcoski PC, Edelbacher M. Perspectives on Elderly Crime and Victimization in the Future. Crime Prevention and Justice in 2030 [Internet]. 2021 [cited 2021 Dec 16];85–106. Available from: https://link.springer.com/chapter/10.1007/978-3-030-56227-4_5
6. Brinkley-Rubinstein L. Incarceration as a catalyst for worsening health. Health & Justice. 2013;1(1):1–17.
7. Jewkes Y, Jordan M, Wright S, Bendelow G. Designing 'healthy'prisons for Women: Incorporating trauma-informed care and practice (TICP) into prison planning and design. International journal of environmental research and public health. 2019;16(20):3818.
8. Pazart L, Godard-Marceau A, Chassagne A, Vivot-Pugin A, Cretin E, Amzallag E, et al. Prevalence and characteristics of prisoners requiring end-of-life care: A prospective national survey. Palliative Medicine. 2018 Jan 1;32(1):6–16.
9. Maschi T, Dasarathy D. Aging With Mental Disorders in the Criminal Justice System: A Content Analysis of the Empirical Literature. International Journal of Offender Therapy and Comparative Criminology. 2019 Sep 1;63(12):2103–37.
10. Petreca VG. Death and Dying in Prison: An Integrative Review of the Literature. Journal of Forensic Nursing. 2021;17(2):115–25.
11. Turner M, Peacock M, Payne S, Fletcher A, Froggatt K. Ageing and dying in the contemporary neoliberal prison system: Exploring the 'double burden' for older prisoners. Social Science and Medicine. 2018;212.
12. Hospice UK. Dying Behind Bars How can we better support people in prison at the end of life? [Internet]. 2021 [cited 2022 Jan 3]. Available from: https://professionals.hospiceuk.org/docs/default-source/Policy-and-Campaigns/huk_dying_behind_bars.pdf?sfvrsn=0
13. NHS England » Resources [Internet]. [cited 2022 Jan 3]. Available from: https://www.england.nhs.uk/eolc/resources/
14. High Income Countries 2021 [Internet]. [cited 2022 Jan 3]. Available from: https://worldpopulationreview.com/country-rankings/high-income-countries
15. Peters MDJ, Godfrey CM, Khalil H, McInerney P, Parker D, Soares CB. Guidance for conducting systematic scoping reviews. JBI Evidence Implementation. 2015;13(3):141–6.

For peer review only

16. Roulston A, McKeaveney C, Anderson M, McCloskey P, Butler M. Deaths in Prison Custody: A Scoping Review of the Experiences of Staff and Bereaved Relatives. The British Journal of Social Work [Internet]. 2021 Jan 1;51(1):223–45. Available from: https://doi.org/10.1093/bjsw/bcaa095
17. Hong QN, Fàbregues S, Bartlett G, Boardman F, Cargo M, Dagenais P, et al. The Mixed Methods Appraisal Tool (MMAT) version 2018 for information professionals and researchers. Education for Information. 2018;34(4):285–91.
18. Joanna Briggs Institute. JBI Manual for Evidence Synthesis 2020 [cited 2022 Jan 3]. Available from: https://jbi-global-wiki.refined.site/space/MANUAL/3318284351/8.4.1+++MMSR+questions+that+take+a+CONVERGENT+INTEGRATED+approach+to+synthesis+and+integration
19. Popay J, Roberts H, Sowden A, Petticrew M, Arai L, Rodgers M, et al. Guidance on the conduct of narrative synthesis in systematic reviews. A product from the ESRC methods programme Version. 2006;1:b92.
20. Lisy K, Porritt K. Narrative synthesis: considerations and challenges. JBI Evidence Implementation. 2016;14(4):201.
21. Tricco AC, Lillie E, Zarin W, O'Brien KK, Colquhoun H, Levac D, et al. PRISMA extension for scoping reviews (PRISMA-ScR): Checklist and explanation. Annals of Internal Medicine. 2018 Oct 2;169(7):467–73.
22. Jean-Baptiste M-C. GLOBAL PRISON TRENDS 2020 Special Focus Pull-out section Alternatives to imprisonment Global Prison Trends 2020. 2020 [cited 2021 Oct 28]; Available from: www.tijthailand.org
23. Ageing prison population - Justice Committee - House of Commons [Internet]. [cited 2021 Oct 28]. Available from: https://publications.parliament.uk/pa/cm5801/cmselect/cmjust/304/30403.htm
24. Old age behind bars: how can prisons adapt to the needs of increasingly elderly populations? - Penal Reform International [Internet]. [cited 2021 Oct 28]. Available from: https://www.penalreform.org/blog/age-bars-prisons-adapt-increasingly-elderly-populations/

For peer review only

Supplementary Material

The preliminary search parameters are English-only published research papers, commentaries and discussions, reviews and policy documents concerning palliative and end-of- life care for the prison population.

#	Query
1	Palliative Care/
2	Terminal Care/
3	Death/
4	Terminally Ill/
5	Hospice Care/
6	Pain Management/
7	Advance Directives/
8	Advance Care Planning/
10	Palliat*.mp.
11	end of life.mp.
12	end of life care.mp.
13	terminal illness.mp.
14	dying.mp.
15	end stage illness.mp.
16	supportive care.mp.
17	symptom management.mp.
18	compassionate release.mp.
19	1 or 2 or 3 or 4 or 5 or 6 or 7 or 8 or 9 or 10 or 11 or 12 or 13 or 14 or 15 or 16 or 17 or 18
20	Prisoners/ or Prisons/
21	Criminals/
22	Jails/
23	Incarcerat*mp.
23	Correctional Facilities/
24	convict*.mp.
25	felon*.mp.
26	offender*.mp.
27	inmate*.mp.
28	penitentiar*.mp. [
29	gaol.mp.
30	secure.mp.

For peer review only

31	20 or 21 or 22 or 23 or 24 or 25 or 26 or 27 or 28 or 29 or 30
32	19 and 31

For peer review only

BMJ Open

Developing a typology of models of palliative care delivery in prisons in high income countries: protocol for a scoping review with narrative synthesis

Journal:	*BMJ Open*
Manuscript ID	bmjopen-2022-060886.R1
Article Type:	Protocol
Date Submitted by the Author:	31-Mar-2022

Complete List of Authors:	GILBERT, Emma; University of Bristol<br>Turner, M; University of Huddersfield, School of Human and Health Sciences<br>de Viggiani, Nick; University of the West of England Bristol, School of Health and Social Wellbeing<br>Selman, Lucy; University of Bristol Faculty of Health Sciences, Palliative and End of Life Care Research Group, Bristol Medical School.

**Primary Subject Heading**:	Palliative care
Secondary Subject Heading:	Health policy, Palliative care, Public health
Keywords:	PALLIATIVE CARE, Adult palliative care < PALLIATIVE CARE, HEALTH SERVICES ADMINISTRATION & MANAGEMENT

SCHOLARONE™
Manuscripts

Developing a typology of models of palliative care delivery in prisons in high income countries: protocol for a scoping review with narrative synthesis

Emma Gilbert[1,2], Mary Turner[3], Nick De Viggiani[4], Lucy E. Selman[1]

[1]Palliative and End of Life Care Research Group, Bristol Medical School, University of Bristol, UK

[2]NHS England, Health & Justice, South-West

[3] School of Human and Health Sciences, University of Huddersfield, Huddersfield, UK

[4] School of Health and Social Wellbeing, University of the West of England, Bristol, UK

Corresponding author: Emma Gilbert emma.gilbert@bristol.ac.uk

For peer review only

Abstract

Introduction

A combination of punitive sentencing practices within ageing populations, compounded by the health challenges faced by people in prison, mean that dedicated palliative care provision within prisons is a pressing requirement. However, evidence about exactly how quality palliative and end of life care is delivered in this environment remains sparse.

This review aims to develop a typology of models of palliative and end of life care delivery within prisons in high-income countries to inform service development and policy.

Methods and Analaysis

We will conduct a scoping review of published studies and grey literature, following the Arksey & O'Malley framework. We will report data on models of palliative and end of life care delivery in prisons in high-income countries. Searches will be undertaken in Medline, EMBASE, CINAHL, Social Sciences Citation Index and PsyINFO for all study types, published from 1st January 2000 to December 2021, and reference lists from key reviews and studies will be screened for additional references. We will also screen grey literature from within other high-income countries using a targeted search strategy. For published reports of original research, study quality and risk of bias will be assessed independently by two reviewers using the Mixed Methods Appraisal Tool. A narrative synthesis of the data will be undertaken, integrating the results of the quality assessment.

Ethics and dissemination

Approval by research ethics committee is not required since the review only includes published and publicly accessible data. We will publish our findings in a peer-reviewed journal as per PRISMA 2020 guidance.

For peer review only - http://bmjopen.bmj.com/site/about/guidelines.xhtml

Protocol registration

The final protocol was registered with the Research Registry on 26.11.2021 www.researchregistry.com

Unique ID number: reviewregistry1260

Strengths and limitations of this study

- This protocol conforms to the Preferred Reporting for Scoping reviews extension (PRISMA) 2020 guidelines.
- The interpretation of 'models of care' escapes clear definition within the research literature, so it is not possible to include it in the search strategy; this information will be extracted, and a typology developed using a pre-piloted data extraction template.
- We adopt a narrative synthesis approach as initial searches suggest that the studies identified will be insufficiently similar in research design and there will be a high volume of grey literature such as policy documents and statutory reports.
- Narrative synthesis will provide an in-depth understanding of the literature on how palliative care is delivered in prisons across high-income countries, informing subsequent research.

For peer review only

For peer review only - http://bmjopen.bmj.com/site/about/guidelines.xhtml

Introduction

This review intends to develop a typology of models of palliative care delivery within prisons in high-income countries. A combination of punitive sentencing practices within ageing populations, compounded by the health challenges faced by people in prison, mean that dedicated palliative care provision is a pressing requirement within many prisons(1) (2). However, evidence about exactly how quality end of life care is delivered in these environments remains sparse (1).

With the largest prison population in Western Europe, the demographic of older people in prison is growing rapidly within England and Wales (prisons are devolved within the UK). People aged 60 and over are the fastest growing age group in the prison estate, which is three times as many as sixteen years ago (3). This trend is visible across Europe - of the 48 prison administrations providing data for the Council of Europe's 2020 SPACE report on prison indicators, 14.8% of inmates were aged 50 or over. Imprisoned individuals living behind bars now represent the fastest growing group in correctional facilities in the UK, as well as Australia, Switzerland, Japan and the USA (4). Table 1 illustrates the percentage increase of older people in prison across some high-income countries; the numbers are expected to increase significantly in coming years (5).

Table 1: Percentage increase of older age people in prison across high income countries

Date range	Country	Age	Percentage Increase	Source of data
2013 -2018	Singapore	60 +	50 %	(6)
2013-2018	S Korea	65 +	45%	(6)
2007-2017	Switzerland	50 +	100%	(6)
1990-2030	USA	55 +	4400%	(6)

For peer review only - http://bmjopen.bmj.com/site/about/guidelines.xhtml

2010-2019	Canada	50+	50%	(6)
2002-2020	UK	60+	243%	(7)
2000-2010	Australia	65+	84%	(8)
2000-2009	New Zealand	50+	94%	(8)

There is evidence of an association between incarceration and poor health outcomes (9). Prisons tend to accumulate individuals who have experienced significant health inequalities, with far greater incidences of mental health and substance misuse disorders, as well as physical health co-morbidities, than the general population (2). These health disparities are often intensified by the environmental challenges of delivering healthcare within the built environment. Ageing buildings which cannot ensure rigorous infection prevention control, cells that lack adequate space for specialist equipment and a regime that imposes limitations on an individual's self-efficacy regarding their own nutrition, physical activity, relaxation and sleep, inevitably affects an individual's ability to cope (10). People in prison consequently face increased morbidity (11). In a 2018 rapid review, the estimated annual prevalence of those requiring end of life care in French prisons was twice as high as the anticipated equivalent expected in the general population, and comparable to a population 10 years' older (11).

Research into palliative care within the penal system is an emerging area, and substantial gaps remain regarding the current nature of provision and best practice models. Recent investigation by the European Association for Palliative Care Task Force for Prisoners addressed some of these through data collection within eight countries, examining palliative care provision, causes of death in custody and the application of early release on compassionate grounds policies (1). This research highlighted the inequitable provision for those either dying or living with a life-limiting illness in prison, as well as the limited potential that current early release policies offer in practice (1). Other salient research has focused on the ethical challenges

For peer review only - http://bmjopen.bmj.com/site/about/guidelines.xhtml

that delivering palliative care within a human rights framework poses within the prison system (12), the experience of terminal illness whilst incarcerated (4), as well as the ‘double burden’ experienced by older people in prison who face additional suffering from the failure of prison healthcare to adequately meet their needs (13).

In the UK, understanding the palliative and end of life care needs for people in prison has gained traction and many prisons have well-coordinated relationships with their local palliative care teams and hospices (14). The publication of the Dying Well in Custody Charter – End of Life Care Ambitions (15) articulated these developments as a set of standards for end of life care in prisons, but there is variation in how the Charter has been applied.

Findings from this review will help ensure that best available evidence informs future provision of culturally relevant, tailored palliative and end of life care and support for people in prison. The evidence from this review will also provide a basis for policy making for health and correctional service procedure and protocol around early release on compassionate grounds and alternative secure accommodation for ageing people in prison and those experiencing life limiting illness.

In accordance with guidelines, our scoping review protocol was registered with the Research Registry on the 26/11/2021 [ID: reviewregistry1260].

Aim

This scoping review aims to map and synthesize the literature on models of palliative and end of life care for people in prison, within prisons in high-income countries. Its objectives are to describe models of service delivery that currently exist in published and grey literature, appraise these models in terms of outcomes and impact, and describe facilitators of and the challenges in delivering different models of palliative and end of life care for people in prison. The synthesis will then consider how the identified models meet the overall intentions of palliative care as defined by the

For peer review only - http://bmjopen.bmj.com/site/about/guidelines.xhtml

World Health Organization (16) , drawing out implications and recommendations for service provision and policy.

Review questions

SPICE framework

Setting – adult prisons both male and female.

Perspective – prison staff, prison volunteers, patients and their family/carers (who have current or prior experience of a family member receiving end of life care in prison).

Intervention – model of palliative care/ end of life healthcare delivery - be it a prison hospice, specialist in-reach palliative care provision to a prison, or another integrated model.

Comparison - qualitative and mixed methods studies are unlikely to have a comparison group; quantitative studies may compare the intervention with usual care or with a comparison/control group.

Evaluation – mapping and describing available models of palliative healthcare delivery in terms of acceptability and usefulness to patients, family/carers and clinicians; outcomes and impact of these models; and facilitators of their implementation.

Primary question: What models of palliative and end of life care for people in prison are described in both the published and grey literature?

Secondary questions:

What evidence exists regarding the outcomes and impact of these models?

What are the facilitators of and challenges in delivering different models of palliative and end of life care for people in prison?

Methods

For peer review only - http://bmjopen.bmj.com/site/about/guidelines.xhtml

Inclusion criteria

Study reports will be included in this scoping review if they meet the following inclusion criteria:

1. Any study reporting new empirical data, regardless of study design.
2. Studies reporting models and mechanisms of palliative and end of life healthcare delivery to the prison population within the UK and other comparable high-income countries.
3. Studies reporting the views and experiences of different models of palliative and end of life healthcare delivery in prison from the perspective of:
    a. People in prison, their families and informal carers (including in bereavement).
    b. Prison staff and volunteers.
4. Studies conducted in high-income countries that are published in English. High-income countries are defined by the World Bank as having a gross national income per capita exceeding $12,056 (17).
5. Studies reported since 1st January 2000 until 11th December 2021.

Exclusion criteria

1. Studies not reported in English.
2. Studies reporting on chronic or life-limiting illness, death and dying within prison and criminal justice contexts where the model of care delivery is not described.
3. Studies about institutions that do not fall under the legal definition of prison (e.g., Immigration Removal Centres), or do not cater for adult people in prison (e.g., Secure Children's Homes).
4. Studies about prison palliative care, where patient/caregiver or staff experiences are reported, but the model of care delivery is not described or evaluated.
5. Studies that focus on components of palliative care provided at specific phases of the disease trajectory and do not describe the overall model of palliative care delivery (e.g., pain management only).
6. Studies from low and middle-income countries.
7. Studies published prior to 1st January 2000.

For peer review only - http://bmjopen.bmj.com/site/about/guidelines.xhtml

Grey literature such as conference abstracts, audits, theses and dissertations, research and committee reports, government reports, policy documents, quality improvement reports and ongoing research will be included if they present relevant empirical data. If there is uncertainty about whether the inclusion criteria are met, or if relevant data cannot be extracted, the authors will be contacted to ask if they can provide additional information and/or further data. If this is not possible the study will be excluded.

Adopting the five stages of the Arksey & O'Malley framework as shown in Table 2 (18), this review aims to identify all relevant literature available on the topic, regardless of study design. This method is especially advantageous for assembling emerging evidence, as well as being suitable for addressing questions that go beyond the scope of effectiveness of an intervention (19). The approach adopts an iterative process of study selection, data collation, synthesis and presentation (20).

Table 2 – Five stages of the Arksey & O'Malley scoping review

Stage of review	Illustration of decisions and issues
Identifying the research question	Theoretical and empirical work describing models of palliative and end of life care delivery to people in prison in the U.K and other high-income countries, with broadly comparable criminal justice systems and approaches to human rights.<br><br>Greater understanding of best practice and challenges and barriers to access.
Identifying relevant studies	Specific search criteria designed with key terms used included palliative care, hospice, end of life, compassionate

For peer review only

For peer review only - http://bmjopen.bmj.com/site/about/guidelines.xhtml

	release, prison, penitentiary, imprisonment, incarceration, jail, custody, advance care planning.
Study selection	Final included studies may include a diverse representation of primary sources; data will be extracted using the JBI Mixed Methods Data Extraction Form following a Convergent Integrated Approach.
Charting the data	Data will be extracted from primary sources, different models of care summarised, best practice and barriers to access identified in a narrative synthesis.
Collating, summarizing and reporting the results	Recommended models proposed with areas for further research and development identified.

This review will build on the five stages of the Arksey & O'Malley framework by including critical appraisal of the quality of published studies, using the Mixed Methods Appraisal Tool (MMAT), version 2018 (21).

Search strategy

The following databases will be searched for English language studies:

Medline and EMBASE in Ovid, CINAHL, the Social Sciences Citation Index and PsyINFO.

Additional hand searches of key journals, screening of reference lists of included studies, citation tracking and input from expert collaborators will supplement the database searches. A further exploration of the grey literature will be conducted

For peer review only - http://bmjopen.bmj.com/site/about/guidelines.xhtml

through searches of key websites (e.g., International Association for Hospice and Palliative Care, the WHO) and key grey literature databases (Google Scholar, ProQuest). Forward searches of included articles will be undertaken in Google Scholar to identify recent citing articles to supplement those identified in database searches.

The Medline search strategy is shown in supplementary material file 1. This strategy will be adapted to the other electronic databases and is available to view in supplementary materials file 2. Any modifications will be reported in the review manuscript. Databases searches were run in December 2021. The expected end date for the review is in September 2022.

Screening and data extraction

Search results from each database will be downloaded and managed in Covidence, an online review management platform (22).

Each title and abstract will be screened against the inclusion/exclusion criteria by one of the review team. A second reviewer will independently screen a sample of 25% of the titles and abstracts. Full text will be reviewed if inclusion is unclear based on title and abstract. Any discrepancies of study inclusion will be adjudicated by a third reviewer. Grey literature will be screened and synthesised separately and will not be subject to the same method of quality appraisal.

EG will extract data using a pre-piloted, customised data extraction form based on the JBI Mixed Methods Data Extraction Form following a Convergent Integrated Approach (23), in Covidence. Data extraction will be reviewed by a second reviewer and modified where needed. Discrepancies regarding data extraction will be resolved by discussion and consensus, and if necessary, include a third reviewer.

Quality assessment

The quality of all included studies published in peer reviewed journals will be assessed independently by two reviewers using the Mixed Methods Appraisal Tool (MMAT), version 2018 (21). This validated tool is appropriate for this review as it can be applied

For peer review only - http://bmjopen.bmj.com/site/about/guidelines.xhtml

to qualitative, quantitative (randomised, non-randomised, and descriptive), and mixed-methods study designs. The tool uses a set of questions specific to study design, converted into four possible scores (worst to best: 25/50/75/100). Disagreements between the reviewers will be resolved through discussion, involving a third reviewer if necessary. No studies will be excluded based on their quality, but the narrative synthesis will reflect on the quality of the identified studies. Grey literature will not be subject to quality appraisal and will be analysed and reported separately.

Evidence synthesis

A narrative synthesis will be conducted to synthesize the findings of the different studies. Due to the potential range of studies that may be included in this integrative review, a narrative synthesis is the most appropriate way to synthesise the findings.

This review will follow the narrative synthesis approach outlined by Popay et al (24). This process will involve developing a preliminary synthesis, exploring relationships within and between the studies, and assessing the robustness of the synthesis overall (19).

Grey literature will be synthesised and described where it adds relevant data to the research topic. The narrative synthesis will move beyond simply summarising the main features of included studies, presenting the data in such a way that it enables investigations into similarities and differences between studies, whilst assessing the data and strength of the evidence (20). The synthesis will be structured around the core models of palliative and end of life care delivery for people in prison. For each model, the following data will be synthesised: effectiveness and impact, facilitators for implementation, challenges and barriers of implementation. Implications for future service delivery, policy and research will be identified.

Patient and Public Involvement

We plan to include two members of the public with experience of end of life care in prisons in the review, inviting them to comment on the narrative synthesis and resulting implications as co-authors on the published review.

For peer review only - http://bmjopen.bmj.com/site/about/guidelines.xhtml

For peer review only

Ethics and dissemination

This scoping review of published/publicly available studies is exempt from ethical approval. The review will be reported as per PRISMA guidance (25), and published in a peer-reviewed journal.

Contributors

EG initiated and designed the review as part of an NIHR pre-doctoral fellowship supervised by LES and NdV. LES, MT and NdV contributed to the design of the protocol. EG drafted the manuscript. All the authors contributed to the revision of the manuscript and approved the final version.

Acknowledgements

The authors would like to thank Sarah Herring, University of Bristol Subject Librarian, for support in developing the database searches and Isla Kuhn, Head of Medical Library Services, University of Cambridge Medical Library, for support in developing the grey literature search strategy.

Funding

This work is undertaken as part of EG's National Institute for Health Research (NIHR) Pre-doctoral Fellowship (Award number NIHR301173). LES is funded by an NIHR Career Development Fellowship. The views expressed in this publication are those of the authors and not necessarily those of the NHS or the NIHR.

Competing interests

None declared.

Provenance and peer review

Not commissioned, externally peer reviewed.

For peer review only

Keywords

Palliative care, Hospice Care, End of Life care, Prisoners, Prisons.

For peer review only

For peer review only - http://bmjopen.bmj.com/site/about/guidelines.xhtml

References

1. Turner M, Chassagne A, Capelas ML, Chambaere K, Panozzo S, Teves CM, et al. Mapping palliative care provision in European prisons: an EAPC Task Force Survey. BMJ Supportive & Palliative Care [Internet]. 2021 Apr 22 [cited 2021 Apr 29]; Available from: https://spcare.bmj.com/content/early/2021/04/21/bmjspcare-2020-002701
2. Stürup-Toft S, O'Moore EJ, Plugge EH. Looking behind the bars: emerging health issues for people in prison. British Medical Bulletin [Internet]. 2018 Mar 1;125(1):15–23. Available from: https://doi.org/10.1093/bmb/ldx052
3. Prison Reform Trusts. Prison: the facts Bromley Briefings Summer 2019 [Internet]. 2019. 1987 [cited 2022 Jan 3]. Available from: http://www.prisonreformtrust.org.uk/Portals/0/Documents/Bromley%20Briefings/Prison%20the%20facts%20Summer%202019.pdf
4. Petreca VG. Death and Dying in Prison: An Integrative Review of the Literature. Journal of Forensic Nursing. 2021;17(2):115–25.
5. Kratcoski PC, Edelbacher M, Kratcoski PC, Edelbacher M. Perspectives on Elderly Crime and Victimization in the Future. Crime Prevention and Justice in 2030 [Internet]. 2021 [cited 2021 Dec 16];85–106. Available from: https://link.springer.com/chapter/10.1007/978-3-030-56227-4_5
6. Jean-Baptiste MC. GLOBAL PRISON TRENDS 2020 Special Focus Pull-out section Alternatives to imprisonment Global Prison Trends 2020. 2020 [cited 2021 Oct 28]; Available from: www.tijthailand.org
7. Ageing prison population - Justice Committee - House of Commons [Internet]. [cited 2021 Oct 28]. Available from: https://publications.parliament.uk/pa/cm5801/cmselect/cmjust/304/30403.htm
8. Old age behind bars: how can prisons adapt to the needs of increasingly elderly populations? - Penal Reform International [Internet]. [cited 2021 Oct 28]. Available from: https://www.penalreform.org/blog/age-bars-prisons-adapt-increasingly-elderly-populations/
9. Brinkley-Rubinstein L. Incarceration as a catalyst for worsening health. Health & Justice. 2013;1(1):1–17.
10. Jewkes Y, Jordan M, Wright S, Bendelow G. Designing 'healthy'prisons for Women: Incorporating trauma-informed care and practice (TICP) into prison planning and design. Int J Environ Res Public Health. 2019;16(20):3818.
11. Pazart L, Godard-Marceau A, Chassagne A, Vivot-Pugin A, Cretin E, Amzallag E, et al. Prevalence and characteristics of prisoners requiring end-of-life care: A prospective national survey. Palliative Medicine. 2018 Jan 1;32(1):6–16.
12. Maschi T, Dasarathy D. Aging With Mental Disorders in the Criminal Justice System: A Content Analysis of the Empirical Literature. International Journal of Offender Therapy and Comparative Criminology. 2019 Sep 1;63(12):2103–37.
13. Turner M, Peacock M, Payne S, Fletcher A, Froggatt K. Ageing and dying in the contemporary neoliberal prison system: Exploring the 'double burden' for older prisoners. Social Science and Medicine. 2018;212.
14. Hospice UK. Dying Behind Bars How can we better support people in prison at the end of life? [Internet]. 2021 [cited 2022 Jan 3]. Available from: https://professionals.hospiceuk.org/docs/default-source/Policy-and-Campaigns/huk_dying_behind_bars.pdf?sfvrsn=0

For peer review only - http://bmjopen.bmj.com/site/about/guidelines.xhtml

15. NHS England » Resources [Internet]. [cited 2022 Jan 3]. Available from: https://www.england.nhs.uk/eolc/resources/
16. Palliative care [Internet]. [cited 2022 Mar 31]. Available from: https://www.who.int/health-topics/palliative-care
17. High Income Countries 2021 [Internet]. [cited 2022 Jan 3]. Available from: https://worldpopulationreview.com/country-rankings/high-income-countries
18. Arksey H, O'Malley L. scoping. Int J Soc Res Methodol. 2005;8(1):19–32.
19. Peters MDJ, Godfrey CM, Khalil H, McInerney P, Parker D, Soares CB. Guidance for conducting systematic scoping reviews. JBI Evidence Implementation. 2015;13(3):141–6.
20. Lisy K, Porritt K. Narrative synthesis: considerations and challenges. JBI Evidence Implementation. 2016;14(4):201.
21. Hong QN, Fàbregues S, Bartlett G, Boardman F, Cargo M, Dagenais P, et al. The Mixed Methods Appraisal Tool (MMAT) version 2018 for information professionals and researchers. Education for Information. 2018;34(4):285–91.
22. How can I cite Covidence? [Internet]. [cited 2022 Mar 23]. Available from: https://support.covidence.org/help/how-can-i-cite-covidence
23. Joanna Briggs Institute. 8.4.1 MMSR questions that take a CONVERGENT INTEGRATED approach to synthesis and integration - JBI Manual for Evidence Synthesis - JBI Global Wiki [Internet]. 2020. 2020 [cited 2022 Jan 3]. Available from: https://jbi-global-wiki.refined.site/space/MANUAL/3318284351/8.4.1+++MMSR+questions+that+take+a+CONVERGENT+INTEGRATED+approach+to+synthesis+and+integration
24. Popay J, Roberts H, Sowden A, Petticrew M, Arai L, Rodgers M, et al. Guidance on the conduct of narrative synthesis in systematic reviews. A product from the ESRC methods programme Version. 2006;1:b92.
25. Tricco AC, Lillie E, Zarin W, O'Brien KK, Colquhoun H, Levac D, et al. PRISMA extension for scoping reviews (PRISMA-ScR): Checklist and explanation. Annals of Internal Medicine. 2018 Oct 2;169(7):467–73.

For peer review only

For peer review only - http://bmjopen.bmj.com/site/about/guidelines.xhtml

Supplementary Material – MEDLINE search strategy

The preliminary search parameters are English-only published research papers, commentaries and discussions, reviews and policy documents concerning palliative and end-of- life care for the prison population.

#	Query
1	Palliative Care/
2	Terminal Care/
3	Death/
4	Terminally Ill/
5	Hospice Care/
6	Pain Management/
7	Advance Directives/
8	Advance Care Planning/
10	Palliat*.mp.
11	end of life.mp.
12	end of life care.mp.
13	terminal illness.mp.
14	dying.mp.
15	end stage illness.mp.
16	supportive care.mp.
17	symptom management.mp.
18	compassionate release.mp.
19	1 or 2 or 3 or 4 or 5 or 6 or 7 or 8 or 9 or 10 or 11 or 12 or 13 or 14 or 15 or 16 or 17 or 18
20	Prisoners/ or Prisons/
21	Criminals/
22	Jails/
23	Incarcerat*mp.
23	Correctional Facilities/
24	convict*.mp.
25	felon*.mp.
26	offender*.mp.
27	inmate*.mp.
28	penitentiar*.mp. [
29	gaol.mp.
30	secure.mp.

For peer review only

31	20 or 21 or 22 or 23 or 24 or 25 or 26 or 27 or 28 or 29 or 30
32	19 and 31

For peer review only

CINAHL search strategy	
S55	S40 AND S54.
S54	S41 OR S42 OR S43 OR S44 OR S45 OR S46 OR S47 OR S48 OR S49 OR S50 OR S51 OR S52 OR S53)
S53	“secure”
S52	“gaol”
S51	“penitentiar*”
S50	“inmate”
S49	“offender”
S48	“felon”
S47	“convict*”
S46	(MH “Correctional Facilities”)
S45	“icarcerat*”
S44	“Jail*”
S43	"Criminal*"
S42	(MH "Prisoners")
S41	"Prison*"
S40	S21 OR S22 OR S23 OR S24 OR S25 OR S26 OR S27 OR S28 OR S29 OR S30 OR S31 OR S32 OR S33 OR S34 OR S35 OR S36 OR S37 OR S38 OR S39
S39	"'compassionate release'"
S38	"'symptom management'"
S37	"'supportive care'"
S36	"'end stage illness'"
S35	"dying"
S34	dying
S33	"terminal illness"
S32	"'end-of-life care'"
S31	"'end-of-life'"
S30	"Palliat*"
S29	(MH "Advance Care Planning")
S28	(MH "Advance Directives+")

For peer review only

S27	(MH "Pain Management")
S26	(MH "Hospice Care")
S25	Terminally ill
S24	(MH "Terminally Ill Patients+")
S23	(MH "Death+")
S22	(MH "Terminal Care+")
S21	(MH "Palliative Care")

For peer review only

EMBASE search strategy	
**#**	**Query**
1	exp palliative therapy/
2	exp terminal care/
3	exp death/
4	exp terminally ill patient/
5	exp hospice care/
6	"pain management".mp.
7	'advance directives'.mp.
8	exp advance care planning/
9	Palliat*.mp.
10	"end-of-life".mp.
11	"end-of-life care".mp.
12	exp terminal disease/
13	"terminal illness".mp.
14	exp dying/
15	"end stage illness".mp.
16	"supportive care".mp.
17	"symptom management".mp.
18	"compassionate release".mp.

19	Palliative care.mp.
20	Terminal care.mp.
21	death.mp.
22	terminally ill.mp.
23	hospice care.mp.
24	exp hospice/
25	advance care planning.mp. or advance care planning/
26	dying.mp.
27	prisoner/ or prison/
28	prison.mp.
29	prisoner.mp.
30	Criminal*.mp.
31	Jail*.mp.
32	Incarcerat*.mp.
33	correctional facility/
34	exp correctional facility/
35	convict*.mp.
36	felon*.mp.
37	exp offender/
38	offender.mp.
39	inmate*.mp.
40	penitentiar*.mp.
41	gaol*.mp.
42	1 or 2 or 3 or 4 or 5 or 6 or 7 or 8 or 9 or 10 or 11 or 12 or 13 or 14 or 15 or 16 or 17 or 18 or 19 or 20 or 21 or 22 or 23 or 24 or 25 or 26

For peer review only

43	27 or 28 or 29 or 30 or 31 or 32 or 33 or 34 or 35 or 36 or 37 or 38 or 39 or 40 or 41
44	42 and 43
45	limit 44 to yr="2000 -Current"

**Social Sciences Citation Index Search Strategy**
**#9 AND**
**#10** and **2000** or **2001** or **2002** or **2003** or **2004** or **2005** or **2022** or **2021** or **2020** or **2019** or **2018** or **2017** or **2016** or **2015** or **2014** or **2013** or **2012** or **2011** or **2010** or **2009** or **2008** or **2007** or **2006** (Publication Years)
**Prion* OR Prisoner* OR Jail* OR Incarcerat* OR "Correctional Facilities" OR Convict* OR felon* OR offender* OR inmate* OR penitentiar* OR gaol** (Topic)
**"Palliative Care" OR "Terminal Care" OR Death OR '"Terminally ill" OR "Hospice Care" OR "Pain Management" OR "Advance Directive*"OR "Advance Care Planning" OR Palliat* OR "end-of-life" OR "terminal illness" OR dying OR "end stage illness" OR "supportive care" OR "symptom management" OR "compassionate release"** (Topic)
**#5 AND #6**
**TS=(Prion* OR Prisoner* OR Criminal* OR Jail* OR Incarcerat* OR "Correctional Facilities" OR Convict* OR felon* OR offender* OR inmate* OR penitentiar* OR gaol OR secure)**
**TS=("Palliative Care" OR "Terminal Care" OR Death OR '"Terminally ill" OR "Hospice Care" OR "Pain Management" OR "Advance Directive*"OR "Advance Care Planning" OR Palliat* OR "end-of-life" OR "terminal illness" OR dying OR "end stage illness" OR "supportive care" OR "symptom management" OR "compassionate release")**
**#1 AND** **#2** and **2000** or **2001** or **2002** or **2003** or **2004** or **2005** or **2006** or **2007** or **2008** or **2009** or **2010** or **2011** or **2012** or **2013** or **2014** or **2015** or **2016** or **2017** or **2018** or **2019** or **2020** or **2021** or **2022** (Publication Years)
**#1 AND #2**

**ALL=(Prion* OR Prisoner* OR Criminal* OR Jail* OR Incarcerat* OR 'Correctional Facilities' OR Convict* OR felon* OR offender* OR inmate* OR penitentiar* OR gaol OR secure)**
**TS=('Palliative Care' OR 'Terminal Care' OR Death OR 'Terminally ill' OR 'Hospice Care' OR 'Pain Management' OR 'Advance Directive*' OR 'Advance Care Planning' OR Palliat* OR 'end-of-life' OR 'terminal illness' OR dying OR 'end stage illness' OR 'supportive care' OR 'symptom management' OR 'compassionate release')**

**PsyINFO Search Strategy**	
1.	Palliative care.mp. or exp palliative therapy/
2.	Terminal Care.mp. or exp terminal care/
3.	exp death/ or Death.mp.
4.	Terminally ill.mp. or exp terminally ill patient/
5.	Hospice Care.mp. or exp hospice care/
6.	'Pain management'.mp.
7.	'Advance* Directive*'.mp.
8.	'Advance* Care Planning'.mp. or exp advance care planning/
9.	Palliat*.mp.
10.	'end of life'.mp.
11.	'end of life care'.mp.
12.	'terminal illness'.mp. or exp terminal disease/
13.	dying.mp. or exp dying/
14.	'end stage illness'.mp.
15.	'supportive care'.mp.
16.	'symptom management'.mp.
17.	'compassionate release'.mp.
18.	1 or 2 or 3 or 4 or 5 or 6 or 7 or 8 or 9 or 10 or 11 or 12 or 13 or 14 or 15 or 16 or 17
19.	Prison*.mp. or exp prison/ or exp prison nursing/ or exp prisoner/
20.	Criminal*.mp. or exp criminal justice/ or exp criminal behavior/

For peer review only

21.	Jail*.mp.
22.	exp incarceration/ or Incarcerat*.mp.
23.	'Correctional Facilities'.mp. or exp correctional facility/
24.	convict*.mp.
25.	felon*.mp.
26.	exp offender/ or offender*.mp.
27.	inmate*.mp.
28.	penitentiar*.mp.
29.	gaol*.mp.
30.	secure.mp.
31.	19 or 20 or 21 or 22 or 23 or 24 or 25 or 26 or 27 or 28 or 29 or 30
32.	18 and 31

For peer review only
